# Breeding of Human Beings—Highest Form of Animal

**Published:** 1909-09

**Authors:** 


### Breeding of Human Beings—Highest Form of Animal.

In the breeding of animals—say race horses for example—it is not enough that the male be swift, strong, of great endurance, sure-footed and possessed of all the qualities of the racer.

Such an animal, to reproduce his like, is bred to a *female of equally fine qualities.*

No one looks for a great product if such an animal is bred to a pitiful little runt of a female; and no one looks for blooded stock, however great the female, if the male be a broncho.

This fact is not the private knowledge of the scientist, of the student of Darwin, of even the practical original experimentalist.

It is known to even horse breeders, making no pretense to a knowledge of biological laws.

It is so universally known as not to have one disputant. Indubitably, it applies to everything in nature, from plants to man.

In plants we have even developed the science of development, of evolution. So with animals—horses, dogs, cows, chickens, etc.

Who shall say, in the face of the evidence, that man is above this iron law of nature?

It explains the "black sheep" and the genius, brothers.

The work and toil and self-development which a sturdy father and excellent mother have had in their lives crops out in the man who makes millions think, or who makes millions of dollars; or who endows the human race with an invention worth countless millions.

The self-indulgence of a successful great man, wedding a woman of means, idleness and freedom from sordid care and (consequently) sympathies, likewise accounts for the offspring of the great man, who, in one son, shows the elements of the greatness of his father and in another son astounds the world by his utter triflingness and sometimes criminality.

It is hard to trace these laws, as they work out in human beings, for we students of them live but one generation; and, then, the students have not that large liberty of experimentation which they exercise in breeding dogs, cattle, horses and plants. But it is desperately certain to cold logic and the coldest hard sense that the laws exist and rule.

This by way of preface to a word or two on the treatment we are according womankind even in this advanced day of light and civilization.

The other day I saw a little girl of fourteen tossing a baseball with all the grace, ease, accuracy, lithe, strength and naturalness of a boy. So pronounced was her skill that my companion remarked it and said: "Why is it that a girl can never throw like a man?"

Just as he said this a dignified dame, powdered and bescented with delicate perfume, fat from overfeeding, rustling in irreproachable silk, who had lifted her lorgnette to regard the little miss, remarked to the woman walking with her, a tall, lank anemic, "How very undignified and wrong!"

"Ah, there's your answer," I said quickly to my friend. "A girl can never throw like a boy because Stupidity, the worst form of inertia, stands against her throwing at all; and back of Stupidity and lending it due reverence is that famous old tyrant, Custom, the bulwark of all the fools alive, the rote-book of every unitiative ass that ever went about in human skin!"

In "Don Juan" Byron says: "Man to man so oft unjust is *always* so to woman!"

How remorselessly and cruelly true this is!

Who has not heard some little lassie, imprisoned by her sex at some time say, with all the earnestness of her soul: "I wish I were a boy!" Who has not heard a woman in despair at the thralldom of sex limitations wail: "I wish I were a man!" In every mood, grave, gay, or resentful, like the girl resting on her plow in the "Prophet of the Great Smoky Mountains"—in every mood and with every variety of impulse, we have heard them wail or shriek or earnestly pray this.

I have never heard a man say he wishes he were a woman!

From the cradle to the grave, under the most fool conventions, we imprison the mothers of the human race.

We laugh at the Chinese for imprisoning the feet of their women to keep them small, and yet we imprison the souls of our women.

If we stopped honestly to ask ourselves a solid reason—one for ten thousand of the indecorous things forbidden by convention, by habit of thought, we could not answer one!

The culminating stupidity is reached when we hold that that red-nosed, corrupt bummer there is fit to vote, to have a voice in government; and that sweet mother there is not!

Why?

He wears breeches and she wears dresses!

Marvelously substantial reason!

Can Bedlam beat it?

There is one truth, however, which, thank God, we may always tie to.

*Evolution corrects all stupidities.*

Nothing lives in convention or in life which has not a *reason* for living.

One after another, the dear stupidities of the world, through all their vast circle, wither and die.

As man advances, human liberty opens like a rose.

As man advances, the position of woman is advanced from its sham queenly slavery to its free queenliness—that of the human being whom we adore and, adoring, are willing to accord everything, trusting to the laws of nature itself for her own wise selection.

Let's get down to a few of the practical bearings of this matter on the intelligence, growth, progress and development of the human race. Turn we to the little miss tossing the ball "like a boy."

I am glad to see our own Sophie Newcomb, presided over by that scholarly, broad, splendid man, diplomat, sociologist, student, scholar—President Dixon—laying an increasing stress on physical exercise and development (I despise the word, culture—"physical culture"; it implies either work or faddism, and exercise of body should be as full of joy as the sense the fresh morning air pours into our lungs).

I am glad to see the girls and young ladies entering into real physical contest games.

Systematic training in gymnasium is fine, like going to lessons systematically in schoolroom; but when did schoolroom work ever compare to the mental awakening given a youngster in a competitive spelling bee where every mind is alert "for blood."

Thrice genteel old ladies of the old school, who think that such rough sport is "horrid" and who need assistance to step across a foot-wide ditch, would not be so short-winded, clumsy, helpless and unhealthy if the part of their young days they spent pronouncing "prunes and prims" and crochetting and looking leaning-willowy had been spent in robust and even rough games of skill and strength—and nature's equipment tells the hereditarily weaker sex how rough the game should be. Nature sets all common-sense limitations.

The spectacle of this advanced thought arriving in the prudish South is a positive inspiration. In the North, the idea has taken such strong root that it is destined to stay and to flourish.

The result is going to be manifest in a decade or so.

Our women are going to be physically more splendid, sturdy, beautiful and the race to be begotten is going to be finer.

Every energy should be directed to the physical development of the boys at school.

Our educational system, slowly but steadily improving, is all wrong.

It should be distinctly directed to three purposes:

The development of the intellect.

The development of the emotional or imaginative faculties.

The development of the physical being. Of what avail is a great brain in a poor little anemic frame? See the big gross dullard who outstrips him in the race of life!

Of what avail is a powerful intellect which has not the emotion, the *imagination* to get beyond the square and compass and see and plan great things which are not?

Up to today, when one considers that marvelous creature man; sees him using a judgment with animals which he does not apply to his own race; he is forced to conclude that at best our ideals of what education is are crass and cheap and miserable even as expedients.

It is just as important that one should train the muscles of the body as of the brain, for what are they but the brain's extension—the executors of the brain's will?

And that child has greater chance for a real, useful education who is made to hear the grand music of the world, to see the beauties of nature and of art, to *create*, too, than the one who is crammed from the "prep." to the graduating year with all the courses of the great college of today.

It is very bad to get into a rut.

Every public schoolhouse should have a gymnasium and place it as a necessity as great as the books in the hands of the children.

Every public school should have a music room and every child be taught music. Real educational advancement will mean that any one of the growing generation of children who can not read music at sight is as ignorant as the person who can not read letters.

The most hopeful sign is that the girls are being given a greater chance, the American continent over.

By and by we shall be civilized.

By and by according to the remorseless laws heretofore rehearsed, American men and women are going to come of real "blooded stock"—the peerage of brain, brawn, health, self-centered independence.—*The Harlequin,* New Orleans.

